# Mastication

**Published:** 1851-04

**Authors:** E. W. Power


### MASTICATION.

BY DR. E. W. POWER.

The special function which the teeth are designed to perform in the animal economy, is the prehension and the mastication of our food. We find them most skilfully adapted to this purpose by the peculiar shape of the different teeth, and their general arrangement in the jaws. The front teeth are bevelled down to a cutting edge, like that of an axe, for the purpose of grasping and dividing the food as it is presented to the mouth, while the molar or jaw teeth possess a broad surface divided into several prominences by transverse indentations, to fit them for breaking down the constitutent texture of the aliment and triturating it until it is properly prepared to be passed into the stomach, where it undergoes the main process of digestion.

For the due performance of the process all physiologists agree that the preparatory mastication of the food and its thorough mixture with the saliva are absolutely essential. It corresponds to the preliminary trituration to which the chemist would submit any solid matter that he might present it in the most advantageous form to a digestive menstruum. It reduces the food to that condition by which it is rendered more susceptible of being acted upon by the solvent powers of the gastric juice.

Mastication thus becomes the initial step in the important function of digestion, without the proper execution of which the ultimate conversion of the aliment into the various tissues of the body will be impeded. For if the food is insufficiently masticated in the mouth, either from haste in eating, or from the want of teeth, the office of the stomach will be overtaxed; hence the *chyme* will be imperfect, the kyle defective, and this impure fluid will be conveyed to the heart, where deteriorated blood will be the result; and as a necessary consequence the functions of *circulation*, *respiration*, and *innervation* be deranged. In this way by disturbing but a single link, the whole chain of dependent processes becomes measurably weakened.

Now we have seen that mastication is the special duty devolving upon the teeth, and when the permanent set of these organs is lost by disease or otherwise, nature has provided no substitute to fill their place. Unlike the other parts of the system, the teeth, from their comparatively low degree of vitality, are destitute of that recuperative energy which is capable of repairing any loss which they may sustain from disease. When the other bones of the body receive an injury, this inherent power repairs the lesion. Not so with the teeth. When they give way beneath the touch of corroding agents, they put forth no intrinsic energy to rebuild their crumbling walls. Hence, when they are once destroyed, no reparation can be effected.

It is said, it is true, that the gum after these organs are removed, assumes a hardened, cartilaginous state which fits it for masticating the food; but a casual glance at the changes which take place in the jaws after the teeth are lost will convince any one that the gum can be at most but a very imperfect substitute. So much absorption invariably takes place in the alveolar process which embrace the roots of the teeth as to prevent the jaws from fairly closing together, and in many instances from even approaching near enough to produce the first movement toward mastication. But even if they did fairly articulate together, they would present but a smooth rounded surface wholly incapable of breaking up the texture of the aliment and mingling it with the saliva, which is the legitimate province of this function.

As nature then, provides no efficient substitute for the teeth, their loss, by impairing mastication, is productive of a deleterous influence, under which the general system must sooner or later inevitably suffer. It may be scarcely perceived at first, and like the evil effects flowing from the violation of any of the great laws of health, the vigorous constitution, before succumbing, may seem to resist it for a length of time, but is not consequently the less certain in final results. The strong man addicted to intemperance, may appear for awhile to defy its influence on his iron frame, yet time invariably develops the fact that his continued vigor and health have been deceptive, but masking over the insidious inroads, which his destructive habit has been making on all his vital energies, so the injurious influence resulting from the imperfect mastication of the food may be alike insidious in its operation, gradually, and it may be imperceptibly to the individual himself, under-mining the strongest constitution, until premature disease and the loss of the general health are the result.

The teeth are also auxiliary to the sense of taste; for the food, or at least a portion of it, from the form in which it is taken into the mouth is not at first appreciable by his sense. It is only as it is comminuted, or separated into small particles that it becomes exposed to the gastatory sensibility. And just in proportion as the food is masticated will the sensation of taste be developed and prolonged. This fact is very plainly evinced, by the difference in our palatal relish, between eating slowly, giving time for thorough comminution and eating rapidly, or bolting our food. The latter mode deprives us of a portion of the enjoyment arising from this sense, and reduces eating almost to a mere mechanical, irksome process which nature has made unavoidable in the maintenance of the animal system.

These organs also aid in speaking. The parts concerned in the primary production of sound are situated beyond the mouth, yet the modification of the voice which constitutes articulate sounds or speech, is mainly effected within the buccal cavity, of whose boundary the teeth compose a part. Hence the difficulty which every one who has lost the anterior teeth, experiences in articulating.

Many of the sounds of the voice are produced by the vibratory action of the tongue against these organs. You will find by trying the experiment, that the letter *t*, and the letters *t* and *h* conjoined, are sounded in this way. Consequently the loss of even a single incisor tooth, produces a difficulty in pronouncing all the words commencing with these letters, and the number of words in our language thus commencing is by no means inconsiderable.

By thus participating in the formation of speech, the teeth extend their instrumentality beyond their physical relations, and become administrative to the mind. Hence their deprivation may diminish the power of that higher faculty, by impairing the utterence of its thoughts, the medium through which it is brought into more direct communication with society.—*Pennsylvania Democrat.*

